# From print to perspective: A mixed-method analysis of the convergence and divergence of COVID-19 topics in newspapers and interviews

**DOI:** 10.1371/journal.pdig.0000736

**Published:** 2025-02-05

**Authors:** Qingqing Chen, Andrew Crooks, Adam J. Sullivan, Jennifer A. Surtees, Laurene Tumiel-Berhalter

**Affiliations:** 1 Department of Geography, College of Arts and Sciences, University at Buffalo, Buffalo, New York, United States of America; 2 Department of Family Medicine, Jacobs School of Medicine & Biomedical Sciences, University at Buffalo, Buffalo, New York, United States of America; 3 Department of Biochemistry, Jacobs School of Medicine & Biomedical Sciences, University at Buffalo, Buffalo, New York, United States of America; 4 Clinical and Translational Science Institute, University at Buffalo, Buffalo, New York, United States of America; University of California Los Angeles, UNITED STATES OF AMERICA

## Abstract

In the face of the unprecedented COVID-19 pandemic, various government-led initiatives and individual actions (e.g., lockdowns, social distancing, and masking) have resulted in diverse pandemic experiences. This study aims to explore these varied experiences to inform more proactive responses for future public health crises. Employing a novel “big-thick” data approach, we analyze and compare key pandemic-related topics that have been disseminated *to* the public through newspapers with those collected *from* the public via interviews. Specifically, we utilized 82,533 U.S. newspaper articles from January 2020 to December 2021 and supplemented this “big” dataset with “thick” data from interviews and focus groups for topic modeling. Identified key topics were contextualized, compared and visualized at different scales to reveal areas of convergence and divergence. We found seven key topics from the “big” newspaper dataset, providing a macro-level view that covers public health, policies and economics. Conversely, three divergent topics were derived from the “thick” interview data, offering a micro-level view that focuses more on individuals’ experiences, emotions and concerns. A notable finding is the public’s concern about the reliability of news information, suggesting the need for further investigation on the impacts of mass media in shaping the public’s perception and behavior. Overall, by exploring the convergence and divergence in identified topics, our study offers new insights into the complex impacts of the pandemic and enhances our understanding of key issues both disseminated to and resonating with the public, paving the way for further health communication and policy-making.

## Author summary

The COVID-19 pandemic has led to diverse experiences influenced by public health measures like lockdowns and social distancing. To explore these dynamics, we introduce a novel ’big-thick’ data approach that integrates extensive U.S. newspaper data with detailed interviews. By employing NLP and geoparsing techniques, we identify key topics related to the pandemic and vaccinations both in newspapers and personal narratives from interviews, and compare the (spatial) convergences and divergences between them. We found that both sources converge to highlight the profound impacts of the pandemic on daily life. However, newspapers provide a macro-level perspective, predominately covering policy, public health efforts and economics, while interviews reveal the nuanced impacts at the micro-level, focusing on personal experiences, emotion and concerns. An intriguing finding is the pronounced concern regarding the reliability of news information from interviews. By showcasing both convergences and divergences in identified topics, our study enhances the understanding of key issues that both disseminated to and resonate with the public, contributing to the development of more effective communication strategies for future public health crises.

## Introduction

The COVID-19 pandemic, hereafter referred to as “the pandemic”, was the first of its kind, in the sense that all people around the world have experienced its impacts. This has resulted in the perennial discussion around physical and mental health problems, social connection disruption, economic loss and increased inequalities which are clear examples of the unprecedented consequences of the pandemic [1–4]. To prevent the spread of COVID-19 in society, governments globally have implemented a range of top-down measures, including border closures, lockdowns, mandates for wearing masks and social distancing [5]. At the same time, from an individual level different bottom-up approaches have also been applied and adopted, such as vaccine uptake, the adoption of top-down measures (e.g., masking) and community engagement to support local actions [5]. These approaches have resulted in a diverse array of community experiences, influenced by individuals’ life situations, places where they were, and the ways in which information was disseminated or communicated by public health officials. This raises the question on how can we learn from those different experiences? Understanding this is crucial for us to move forward and be relatively more proactive in our response to new emerging public health crises as opposed to being reactive. To this end, this paper aims to uncover the key topics surrounding the pandemic that have been disseminated *to* the public and delves into major discussions about the pandemic experiences *from* the public, as well as investigating the potential connections between these two aspects.

Looking back in history, one of the earliest mediums of dissemination can be traced back to early Roman times, where news was disseminated via handwritten papal bulls and pasquinades that appeared on Roman doors and walls [6]. However, this method for documenting information slowed its dissemination. This was radically changed in the first half of the 14th century, when the German inventor Johannes Gutenberg introduced the printing press in Europe [7]. The printing press drastically reduced the cost and time of producing written material, thus significantly increasing their availability and dissemination [8]. Through printing technology people could, for the first time, communicate more widely or spread ideas in a relatively easy manner. Emerging from this technological advancement, newspapers have evolved to become one of the most popular forms of mass media worldwide, engaging the public with diverse topics and interests. With the advent of the internet and growth of the world wide web, traditional printed newspapers are increasingly transitioning to digital formats. This shift has relaxed the constraints of print media, allowing information to reach a broader audience globally and has become a mainstay in modern society. Compelling statistics has shown that about 70% of households in the United States with an income over $100K are reported to be newspaper readers, highlighting the continued relevance and influence of this medium [9].

Along with increasing digitization of news is the creation of digitized historical newspaper databases. This type of data is now referred to as “big” data, as it allows for the collection of millions of newspapers at large scales and over long-term periods, opening new opportunities for the exploration of complex societal issues as depicted in them [10]. The utility of this type of data is further amplified by advanced data-driven methodologies, such as Natural Language Processing (NLP) and text analytics, which can unlock valuable insights that were previously unattainable manually. In public health research, for example, Liu et al., [11] utilized topic modeling to investigate health communication patterns in newspapers during the COVID-19 pandemic. Chen & Zhu [12] employed text mining techniques to quantify uncertainties in China’s health policies based on newspapers, shedding light on the volatility and risks associated with the health environment and policy landscape during the pandemic. Similarly, Almomani et al., [13] conducted thematic analysis of newspapers to identify discussions surrounding the issue of fake prescription medicines sold online, highlighting the risks of such online purchases and the influential role of news media in shaping consumer purchasing decisions. These examples, along with other studies [e.g., 14–19], demonstrate the critical role of newspapers in public health communication and education, and their potential to reflect and manage public issues and infodemics [e.g., 20–21].

While newspapers serve as a valuable source of public health information, it is crucial to point out that it is only a “one-way” communication mechanism, where information is transferred in one direction from the sender to the receiver (e.g., authoritative sources communicating their views to the general public) [22]. That being said, it fails to incorporate individuals’ perspectives [23], therefore potentially missing out on crucial social realities. This limitation is particularly significant in the context of our study on the pandemic, which raises the critical question: “*Do newspapers reflect the actual experiences of the public during the pandemic*?” An in-depth understanding of individuals’ perceptions regarding the pandemic experience is crucial and this is where the “thick” data comes into play. “Thick” data, in contrast with “big” data, refers to smaller, more detailed data that provide granular and specific knowledge [24]. Although the sample size is small, “thick” data encompasses the qualitative aspects of the human experience, often collected through ethnographic studies, and is instrumental in revealing underlying human emotions, stories, motivations, and experiences. This rich, human-centric perspective is vital for a comprehensive understanding of the pandemic’s impacts. As such, we would argue that integrating “big” data from newspapers with “thick” data from individual experiences offers a more holistic approach. The blending of “big-thick” data allows for deeper insights into the varied impacts, experiences and concerns related to the pandemic, enabling a more complete and nuanced understanding of its multifaceted nature.

Previous studies have combined newspapers and interviews to investigate public health issues, for example, Vilar-Compte et al. [25] used a combination of newspaper articles and interviews to examine how donations and government actions influenced Mexico post-earthquake breastfeeding practices. In another study, Katikireddi & Hilton [26] used a similar mixed-method approach to analyze the policy process behind the implementation of Minimum Unit Pricing (MUP) for alcohol in Scotland. However, these investigations relied on relatively small samples of newspapers. Such samples, while informative, may not provide the broader scale typically associated with “big” data and are limited in their geographical breadth. Furthermore, these studies primarily relied on qualitative analysis of their mixed data, not leveraging the advantages of advanced quantitative techniques. To the best of our knowledge, our work represents the first unique effort to conduct a large-scale analysis of newspaper data alongside interview data, employing geospatial quantitative analysis. This approach allows us to examine a broader geographical and thematic variability in topics related to the pandemic and vaccination, offering insights into the nuanced connection between public discourse and individual experiences during health crises.

Specifically, in this study, we take the promising synergy of “big-thick” data to unpack the discourse surrounding the pandemic and subsequent vaccinations, both in newspapers and individual narratives. This approach aims at understanding the multifaceted impacts of the pandemic, represents a novel integration and comparison that, to our knowledge, has not been previously undertaken in the context of the COVID-19. Our specific objectives are to answer the following research questions: 1) What topics related to the pandemic and vaccination are presented in newspapers and discussed among individuals in certain communities? 2) Is there any geographical variability in these identified topics? and 3) What are the convergences or divergences of topics between the two data sources?

To explore these questions, we selected the United States as a test case and utilized a large dataset comprising 82,533 newspaper articles collected from January 2020 to December 2021. This dataset is supplemented by “thick” data from semi-structured interviews and focus groups (referred to as “interviews”). We began with developing a systematic framework for geoparsing and geocoding, assigning geographical information to each newspaper article by leveraging NLP techniques and the GeoName geographical database. We then applied topic modeling, a commonly used method for information extraction [27], to identify key topics in both newspapers and interviews. These topics were contextualized with corresponding keywords and cross-compared to reveal areas of convergence and divergence. Additionally, we visualized the identified topics at various scales to highlight potential geographical variations.

In doing so, this study not only uncovers the broader topics presented in newspapers but also sheds light on the nuanced themes reflecting community reactions and knowledge about the pandemic. The synergy of “big-thick” data, combined with advanced text analysis techniques, enables us to capture a richer nature of the impacts of the pandemic and contribute to a systematic exploration of health-related discourse. This line of inquiry has the potential to further advance health communications by improving our understanding of the mechanisms through which information is disseminated in mass media and what individuals’ perceptions are, therefore enabling us to be better prepared for future outbreaks of infectious diseases.

In the remainder of the paper, we will demonstrate the utilized methodology, which includes data collection, geoparsing, and topic modeling (section “Methodology”). After that, we will summarize the identified topics from both newspaper and interview data, followed by the comparisons of convergence and divergence between the two to uncover the multi-faceted nature of impacts of the pandemic (section “Results and Discussion”). The key findings and how our work contributes to the current literature will be discussed in the Conclusion section.

### Methodology

**Fig 1:**
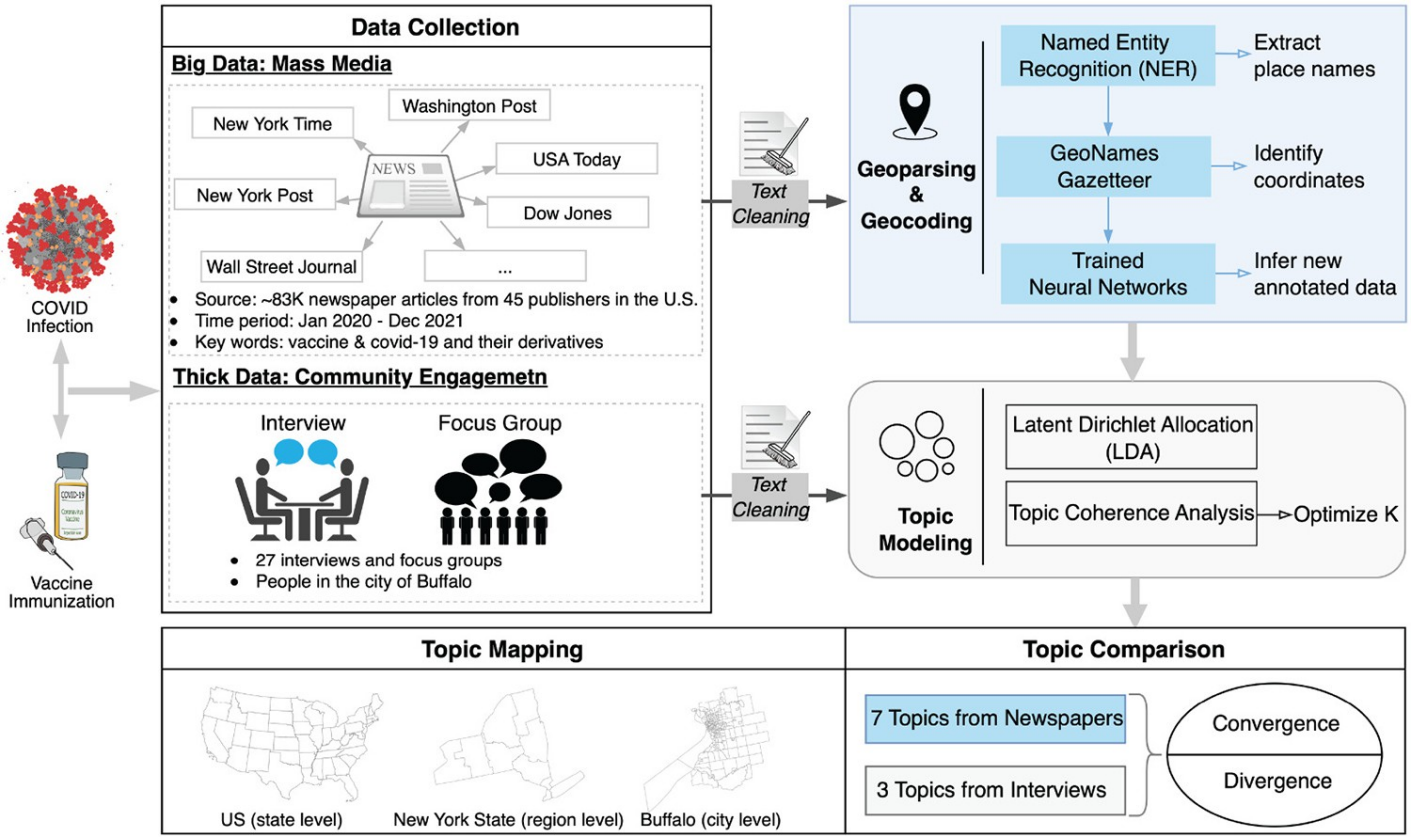
An overview of the research workflow. The base map layer is created using TIGER/Line shapefile provided by the U.S. Census Bureau, available from DATA.GOV. The icons in the figure are sourced from openclipart.org.

In order to identify the convergence and divergence of COVID-19 topics discussed in newspapers and people’s actual perceptions regarding the pandemic, we carried out a mixed-method analysis, combining quantitative and qualitative data. Fig 1 gives an overview of the research workflow and in what follows we provide more details.

### Data collection

#### Quantitative data

**Fig 2:**
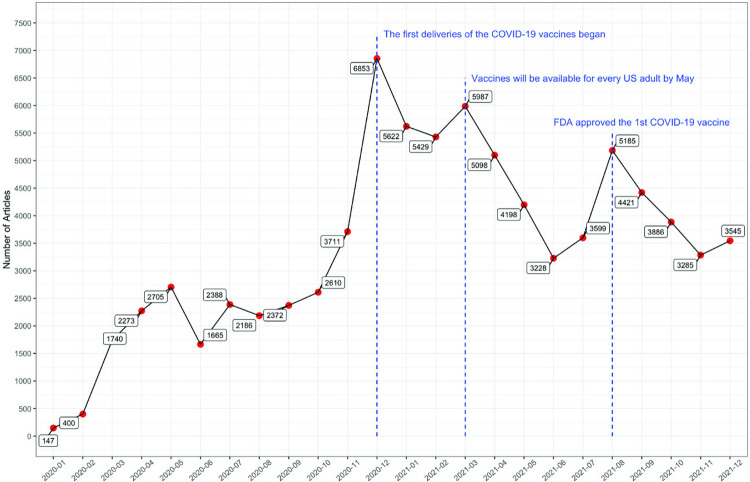
The monthly distribution of collected articles in the United States from January 2020 to December 2021.

To operationalize our mixed-method approach, we first started with the collection of newspaper articles within the United States from January 2020 to December 2021. This data was sourced through Factiva, which is a comprehensive database offering extensive news stories, periodical articles, and financial data from a multitude of global sources. Specifically, our search strategy entailed using key terms associated with COVID-19 vaccinations, such as “vaccine”, “vaccination”, “Pfizer”, “Moderna”, “Novavax”, and “Johnson/J&J”, in conjunction with terms for COVID-19, including “covid”, “covid-19”, “covid19”, and “coronavirus”. This approach ensured the selection of newspapers specifically relevant to COVID-19 vaccination discussions. We then filtered the results to include only English language newspapers, yielding a substantial dataset of 82,533 newspaper articles. Subsequently, these newspaper articles underwent a sequence of preprocessing tasks to refine the dataset for analysis, including removing non-word characters, Unicode characters, hashtags, URLs, stop words, as well as the replacing contractions to their full-word equivalents (e.g., US to United States). This step is crucial to eliminate textual noise and enhance the effectiveness of the subsequent analysis. Fig 2 illustrates the monthly distribution of these collected newspaper articles. To provide context, we included vertical blue dashed lines in this figure, which represent significant COVID-19 vaccine milestones as released by the U.S. Department of Health and Human Services [28]. This visual representation indicates that the collected newspaper dataset effectively mirrors the broader vaccination narrative, capturing key announcements and developments. Further details regarding the sources and publishers of these newspapers are available in S2 and S3 Figs.

#### Qualitative data

In addition to collecting newspaper articles, we employed semi-structured interviews and focus groups to understand the public’s experiences with COVID-19. This was a collaborative effort from the researchers at University at Buffalo and substantial community input from Patient Voices and our Community Advisory Board members. This input shaped the interview guide, ensuring it resonated with what the community experienced during the early stages of the COVID pandemic (i.e., between January and March 2020). To identify potential red flags observed in communities, this guide concentrated on initial community responses, communication styles and modes, as well as the broader implications of these communication strategies. In order to describe our participants, we developed a comprehensive demographic survey. This survey captured individual-level data, and, when relevant, organization information for those representing community-based organizations. A critical aspect of this survey was the identification of high-risk and underrepresented groups, ensuring a representative sample with diverse perspectives. As such, the survey was designed to thoughtfully reflect the intersectionality of communities presented within our participants base.

Our recruitment strategy for the interviews was both broad and purposeful. We implemented a variety of recruitment methods to ensure a rich tapestry of perspectives. More specifically, we extended invitations through purposefully inviting potential participants through our networks that represented diverse communities, either personally or through their professional role. This approach fostered a “snowballing” effect, where participants recommended others with unique viewpoints. Ultimately, key contributors included the Patient Voice Network, a grass-roots community partner representing mostly low-income, Black residents of the city of Buffalo, as well as members of the Buffalo Research Registry–a broader Western New York registry of community residents interested in research participation- residing within specific ZIP codes within the city of Buffalo. Invitations to participate were disseminated through local community partners like libraries, local networks, community-based organizations, and at outreach events. In the end, we recruited a total of 27 people across different communities. This study was conducted under the ethical guidelines and approval of the University at Buffalo Institutional Review Board (UBIRB). The study was reviewed and approved by UBIRB with the approval number: MOD00013577. The formal consent was obtained verbally from all participants before their involvement in the interviews. S1 Fig gives an overview of the distribution of demographic characteristics of these participants.

### Geoparsing

Given that the discussion in newspapers may show regional variations, incorporating a spatial perspective is crucial to analyze news coverage and track specific place-based discussions. However, one primary challenge in our dataset is the absence of direct geographical information. To address this, we implemented geoparsing, a toponym resolution process of identifying and resolving place names within texts to their geographic coordinates. To do so, we utilized the Mordecai library–a full-text geoparsing Python library [29]. More specifically, it involves two main steps–Named Entity Recognition and Geocoding–which we will illustrate in the following sections.

#### Named Entity Recognition (NER)

**Fig 3:**
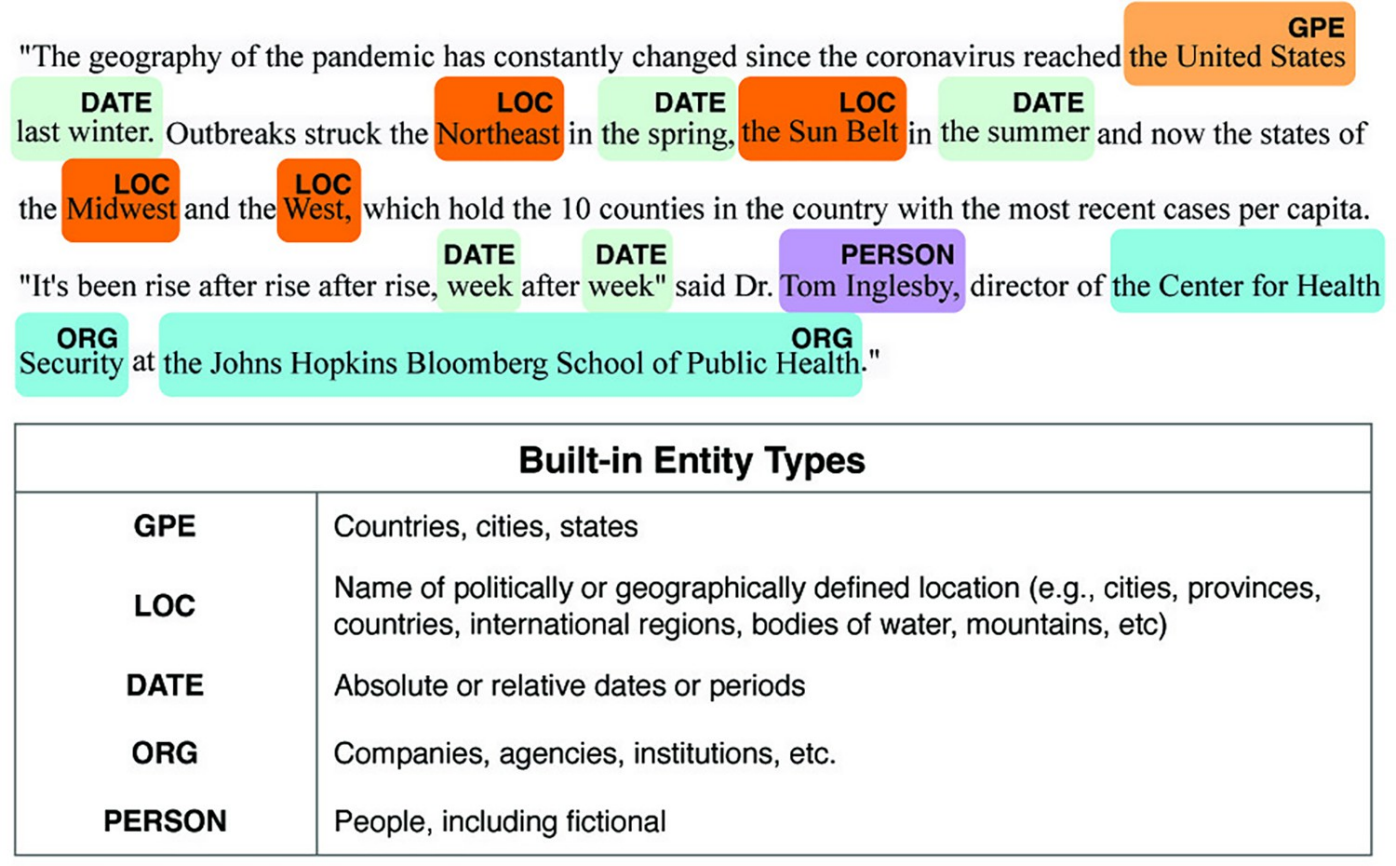
An example of identified entities labeled with predefined entity types.

Named Entity Recognition (NER) is an information extraction technique that identifies and categorizes entities in text into predefined categories, such as persons, locations, organizations, and time expressions. Mordecai uses spaCy’s NER model for identifying place names, which provides a highly efficient statistical system for NER. Its default model can recognize a diverse range of entities, ranging from persons to locations. Fig 3 gives an example of the identified entities in sentences from a newspaper article, highlighting five entity types: *GPE*, *LOC*, *DATE*, *PERSON* and *ORG*. The accompanying table provides descriptions of each entity, with a complete list of spaCy’s built-in entity types available in Appendix S1 Table. We applied NER to each newspaper to label their entities (to see an example of our validation of our method readers are referred to S1 Table).

#### Geocoding

**Fig 4:**
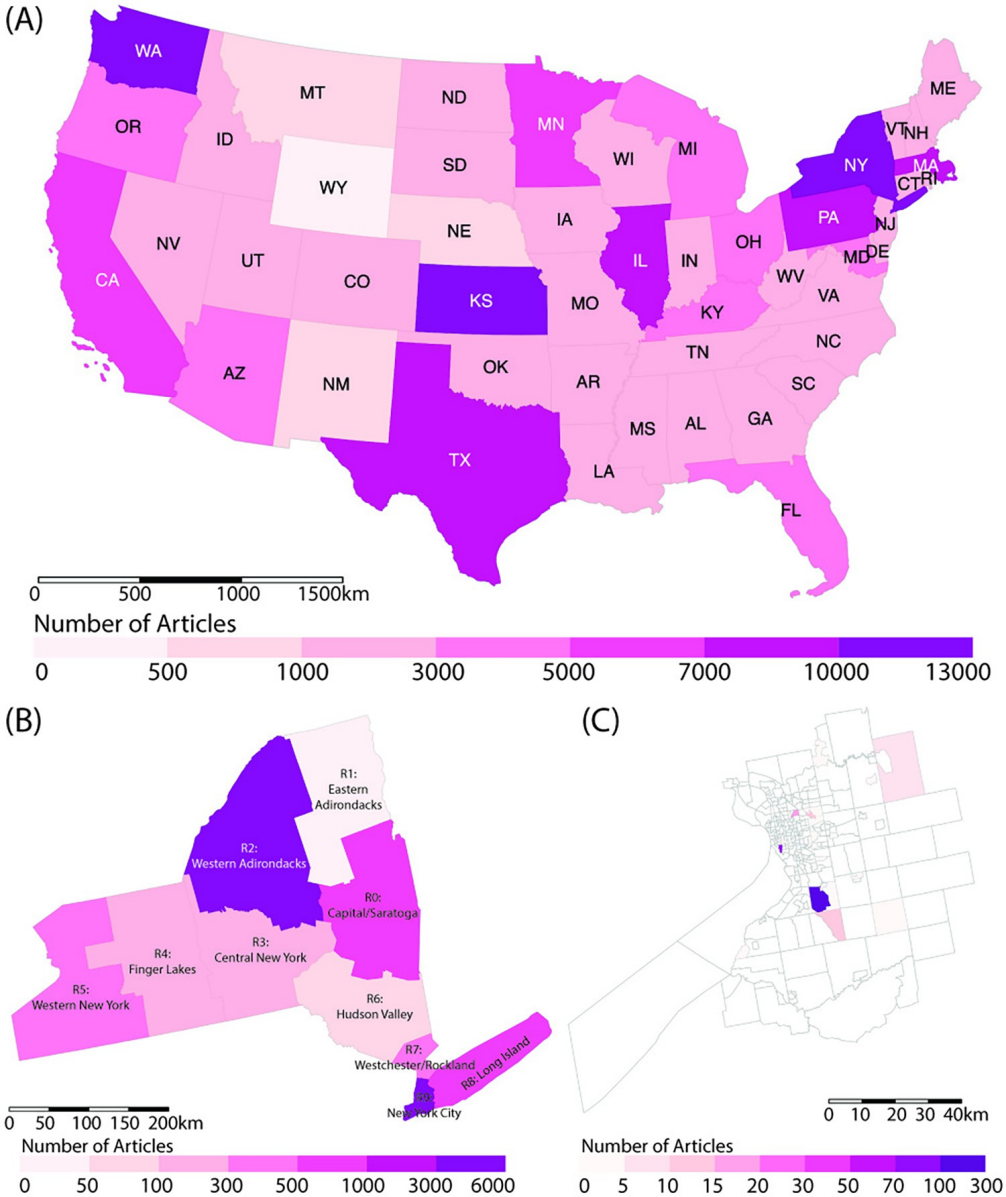
The spatial distribution of newspaper articles by different scales. (A) National level; (B) State level; (C) City level. The base map layer is created using TIGER/Line shapefile provided by the U.S. Census Bureau, available from DATA.GOV.

To geocode the extracted place-related entities (e.g., GPE and/or LOC) identified during the NER process, we utilized the GeoNames index [30], a widely used global dataset of place names [31]. Renowned for its expansive global coverage, GeoNames aggregates data from authoritative sources such as the United States National Geospatial-Intelligence Agency. This database is continually updated through a rigorous quality assurance process, ensuring its accuracy and reliability [32]. Its efficacy and reliability in delivering precise geospatial analyses have been further validated in a recent comprehensive survey on geocoding algorithms [33]. Following the GeoNames indexing, the most likely match is determined using a neural network trained on labeled English texts. Matches surpassing the default probability cutoff (e.g., 0.6) yield a “geo” dictionary which records the geographical coordinates of the matched place names. We applied this process to each newspaper article, geocoding all identified place names. Newspaper articles without identifiable place names were excluded, resulting in 63,553 newspaper articles (97.7% of the initial collection) within the United States. Fig 4 gives an overview of spatial distribution of newspaper articles at different scales.

### Topic modeling

Following the geoparsing phase, our analysis progressed to discerning the convergence and divergence of COVID-19 topics in newspaper discussion compared to public perceptions across regions. To do this, we employed topic modeling techniques, specifically using the Latent Dirichlet Allocation (LDA) model. LDA, a form of probabilistic modeling as noted by Blei et al., [34] is adept at identifying topics within a collection of documents by grouping words with similar meanings. This method treats documents as composites of various topics and each topic as a mixture of words. We chose LDA because of its ability to yield highly interpretable topics, which we would argue is crucial for creating meaningful topic labels beyond just statistically relevant word groupings.

The process of topic modeling begins with transforming unstructured text into a machine-readable format. Specifically, full texts are tokenized into unigrams (single words) and bigrams (two-word phrases). Following tokenization, lemmatization is applied to standardize tokens to their base forms. These tokens are then converted into a Term-Document Matrix (TDM), a sparse matrix representing words and documents. The TDM serves as the input for the LDA model to identify topics. To run the LDA model, we need to manually choose the number of topics, denoted as “k”. In order to determine the optimal k, we utilized the “textmineR” R package [35] for coherence analysis, which evaluates the probabilistic coherence of each topic for a given k. A higher coherence score indicates more associated words per topic, thus, better quality. After testing various k values, we found the optimal number of topics to be k = 7 for newspaper articles and k = 3 for interview data. Consequently, these values were used for running the LDA models on the respective datasets. S4 Fig illustrates the points where the highest coherence scores were achieved, thereby providing an empirical basis for our choice of topic numbers. For reproducibility and replicability purposes, all source code we generated can be found at: https://figshare.com/s/339b1c0d059c189dd6a4.

## Results and discussion

Building upon the methodology, we now turn to discussing the results of our study. We will first focus on the identified topics derived from both newspaper articles and interview discussions. This will be followed by exploring the discrepancies and similarities between these two distinct data sources. This comparative analysis helps reveal the diverse perspectives and approaches in how the mass media and the public have perceived and responded to the COVID-19 pandemic.

In terms of the newspaper articles, we identified seven key topics, each with its distinct importance as quantified by their respective probabilities (see Table 1). To contextualize these topics, we present a summary for each topic based on the corresponding keywords. These range from public health issues to economic impacts and social consequences, reflecting the diverse nature of the pandemic coverage in the mass media. A brief summary of each topic is provided to illustrate their significance and scope in the broader mass media narrative of the pandemic.

**Table 1:** Ordered rank of identified topics by percentages from newspaper articles.

Rank	Topic	Percentage	
1	Topic N1: Public Health & Vaccine Deployment	0.1918	vaccinate, disease, dose, health, people, vaccination, covid, virus, vaccine, coronavirus, dr, infection, report, death, datum, patient, disease control, center disease, hospital, prevention, control prevention, variant, control, cdc, shot, administration, drug, official, center, week
2	Topic N2: Community Health & Vaccination	0.1616	vaccinate, vaccination, county, health, dose, resident, receive, department, worker, shot, care, official, covid, employee, mandate, hospital, community, include, federal, staff, covid_vaccine, medical, require, administer, eligible, week, health care, appointment, health department, clinic
3	Topic N3: Societal Impacts	0.1508	vaccine, covid, people, covid_vaccine, pandemic, time, coronavirus, business, week, health, day, president, receive, school, article, city, support, provide, month, family, continue, life, law, office, change, write, story, court, public, fund
4	Topic N4: Community Responses	0.1354	health, virus, life, care, disease, american, family, hospital, child, medical, university, community, feel, country, unite, spread, study, doctor, coronavirus, time, live, patient, expert, social, school, person, public health, learn, risk, question
5	Topic N5: Safety Measures	0.1205	mask, wear, county, people, city, wear mask, business, distance, reopen, restaurant, school, restriction, resident, house, social, return, indoor, student, week, travel, spread, mandate, close, social distance, plan, feel, pandemic, percent, governor, district
6	Topic N6: Presidential Election	0.1203	trump, biden, president, house, republican, administration, white_house, white, american, democrat, government, official, federal, country, vote, election, senate, political, democratic, unite, leader, joe, washington, nation, campaign, congress, governor, donald, law, court
7	Topic N7: Economic Impacts & Corporate Strategies	0.1196	company, market, stock, business, report, include, industry, government, employee, expect, coronavirus, plan, datum, federal, share, fund, price, drug, economy, rate, accord, global, economic, increase, month, fall, executive, rise, sale, firm

### Topic N1: Public Health & Vaccine Deployment

This topic focuses on vaccine deployment and disease control. Specifically, it spans a broad spectrum of keywords, such as “vaccinate”, “dose”, “vaccine”, “infection”, “death” and “patient”, which indicates a comprehensive discussion on vaccination strategies, the nature of the virus as well as its variants. Additionally, significant emphasis of this topic is placed on the roles of health officials, the CDC, and healthcare centers in managing the pandemic, highlighting key aspects like drug administration and the crucial task of disease prevention and control.

### Topic N2: Community Health & Vaccination

The second topic shows the granular aspects of vaccination administration and its impact on community health. It covers the distribution of vaccine doses, the role of health departments and workers in the vaccination process, as well as the implementation of health mandates. The keywords such as “hospital”, “community”, “health care”, “clinic”, underscore the multi-layered strategy for public health management, which highlights the collaboration between diverse healthcare entities and officials.

### Topic N3: Societal Impacts

This topic captures the profound societal impacts of the pandemic. Keywords such as “vaccine”, “pandemic”, “school”, “family”, “business” emphasize the extensive changes experienced in both public and private life. It also highlights the governmental and legal responses, as indicated by terms like “president”, “law”, “office”, “court”. The inclusion of “fund” and “support” points to the important roles of public funding and health support systems in addressing the challenges of the pandemic. Overall, this topic underscores the intertwined relationship between health policies, societal adaptation and public response during the pandemic.

### Topic N4: Community Responses

The next topic focuses on the challenges faced by healthcare systems and the responses across communities. It looks at how families, medical institutions, universities, and communities responded to the spread of the virus, highlighting the experiences and feelings/emotions of patients, the impacts on children and schools and the role of public health experts.

### Topic N5: Safety measures

This topic contains information about the safety measures implemented during the pandemic. It includes discussions on mask mandates, social distancing and the reopening of business and schools. Keywords such as “reopen”, “restaurant”, “travel” implies the challenges and strategies involved in resuming normal activities but at the same time maintaining public safety. This topic also reflects on the impact of these measures on people’s daily lives, and the actions taken by local governments.

### Topic N6: Presidential election

The sixth topic details the complexities of the U.S. presidential election during the pandemic. It focuses on the presidential candidates, “Trump” and “Biden” and their respective campaigns. Specifically, it covers the operational aspects of the election, notably “voting” processes and “campaigning” tactics under the unique pandemic conditions. This topic is interesting especially for understanding how the unprecedented circumstances of the pandemic may have intersected with or influenced the political dynamics, the outcomes of the election as well as the public’s voting behaviors.

### Topic N7: Economic Impact & Corporate strategy

The final topic anchors on the economic impact of the pandemic and the corresponding strategies employed by corporations. This discussion ranges from market trends and stock market volatilities to the broader economic challenges under the impact of the pandemic. It also includes the discussion around governmental interventions as indicated by keywords like “federal”, “share”, “fund”, and “executive”. These terms underscore the relationship between government actions and business responses in a time of global economic uncertainty during the pandemic.

In addition, the results reveal that three topics have slightly higher coverage probabilities: “Topic N1: Public Health & Vaccine Development (19%)”; “Topic N2: Community Health & Vaccination (16%)” and “Topic N3: Societal Impacts (15%)”. These topics underscore their crucial importance in addressing the immediate challenges of the pandemic, aligning with the media’s role in prioritizing topics that engage public interest. However, despite the highlighted emphasis on these key topics, the overall distribution of media coverage across all topics remains relatively balanced, where most topics are covered at around 12–13% (as shown in Table 1). This balanced coverage allows us to capture a wide range of impacts during the pandemic, offering a multifaceted narrative of this unprecedented crisis.

**Fig 5:**
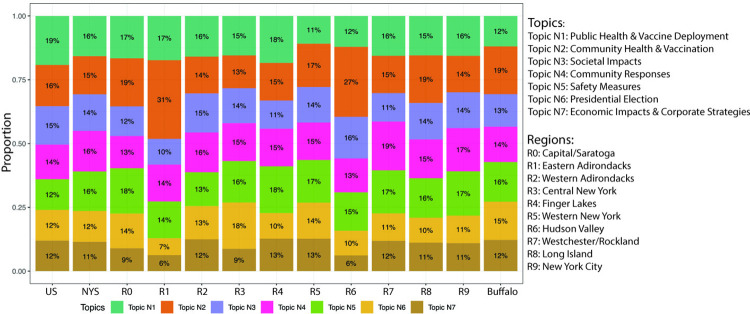
The distribution of identified topics by different scales.

**Fig 6:**
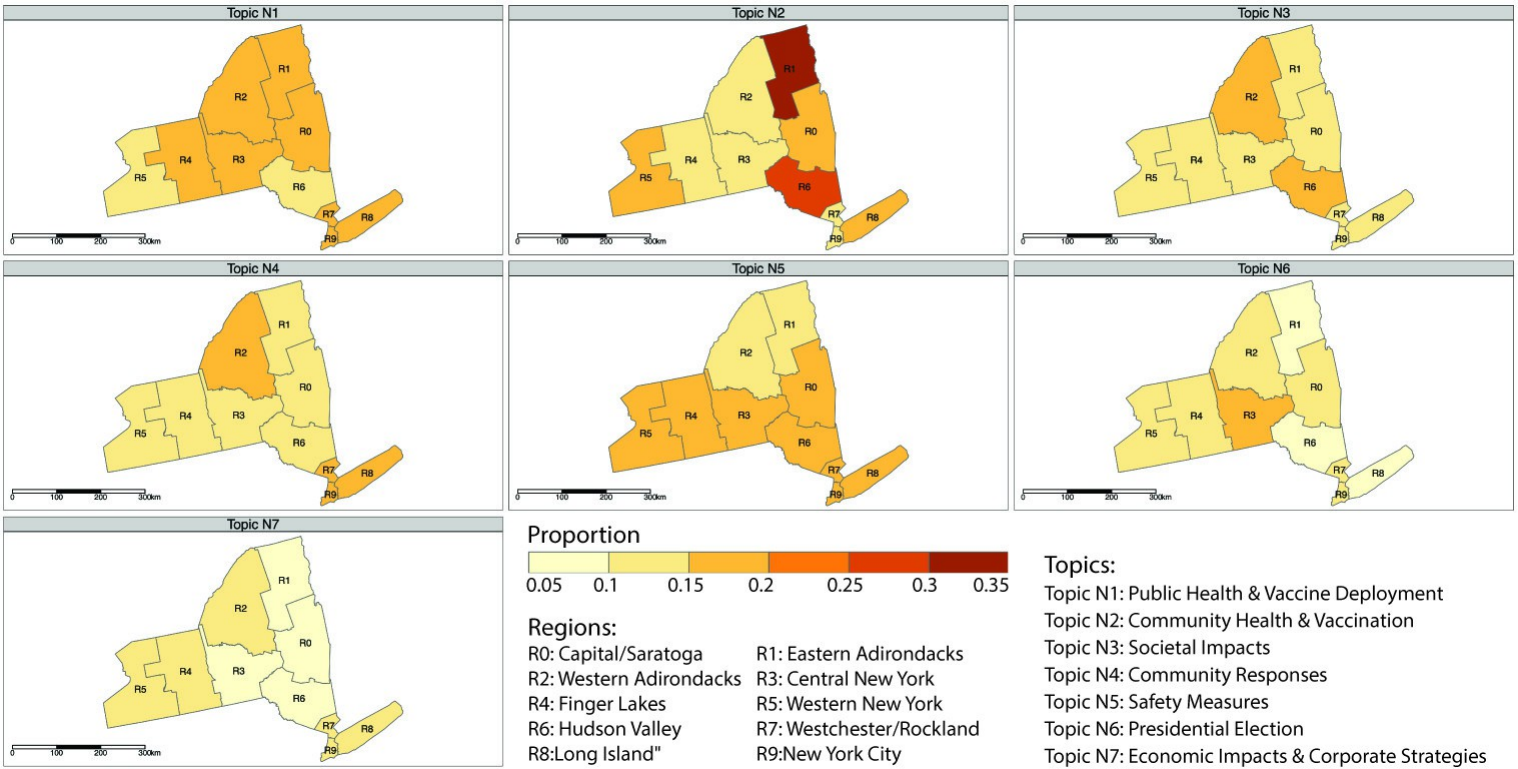
The spatial distribution of identified topics across different regions in New York State. The base map layer is created using TIGER/Line shapefile provided by the U.S. Census Bureau, available from DATA.GOV.

To further investigate these topics over space, we narrow our focus to a more localized context. Specifically, we zoomed into the spatial distribution of these topics within the 10 regions of New York State (denoted as R0-R9) and the city of Buffalo (see Figs 5 and 6). By comparing the general trends observed nationally with local variations, we can gain insights into how regional factors and local contexts may shift the media coverage, which is crucial for understanding the geographic variability in pandemic coverage at a more granular level.

Fig 5 presents an overview of the variance in topics across different scales (e.g., national, state and regions). More specifically, Topic N1 (“Public Health & Vaccine Deployment”) has the highest proportion at the national level (19%), followed by R4 (Finger Lakes) region in New York State. This suggests a nationwide priority on public health issues. Topic N2 (“Community Health & Vaccination”) peaks in the R1 (Eastern Adirondacks) and R6 (Hudson Valley) regions, with a percentage of 31% and 27%, respectively. In contrast, these regions show less emphasis on Topic N5 (“Presidential Election”) and Topic N6 (“Economic Impact & Corporate Strategy”), each accounting for less than 10% of coverage. This pattern demonstrates a regional prioritization of community health over political topics, possibly due to specific local conditions or developments.

The distribution of Topic N3 (“Societal Impacts”) and Topic N5 (“Safety Measures”) is relatively uniform across different areas, with variations within a 5% range. This phenomenon indicates a consistent level of emphasis on societal impacts of government mandates across New York State. In terms of topic N4 (“Community Responses”), R7 (Westchester/Rockland) region shows the highest proportion (19%). As mentioned earlier, this topic highlights healthcare challenges and community responses, especially regarding patient experiences and the impacts on children and schools. The reason for this focus of the region warrants further exploration. Moreover, it is interesting to notice that the R3 (Central New York) region exhibits a notable emphasis on Topic N6 (“Presidential Election”) while comparatively less on Topic N7 (“Economic Impact & Corporate Strategy”).

Turning to the qualitative data collected from interviews, we identified three key topics—“News Broadcasting & Trust”, “People & Everyday life” and “Mandates & Mental Health”, as detailed in Table 2. Each topic was named based on its corresponding keywords, accompanied by a brief summary for clarity.

**Table 2:** Ordered rank of identified topics by percentage from interviews.

Rank	Topic	Percentage	Top important words
1	Topic I1: News Broadcasting & Trust	0.4639	channel, trust, watch news, local news, time, attention, fauci, department health, precaution, CNN, true, honest
2	Topic I2: People & Everyday life	0. 2824	kid, child, husband, son, parent, risk, expose, clean, travel, fever, physician, symptom, condition, respiratory, positive, wear mask, screen
3	Topic I3: Mandates & Mental Health	0.2536	service, food, vaccine, house, staff, county, access, community, hear, understand, health department, mental, virtual, social, provide, phone, answer, datum, prepare, vaccinate

### Topic I1: News Broadcasting & Trust

This topic focuses on the sources of information about the pandemic and vaccination, which aligns with the interview questions (i.e., interview question orientated). The participants’ responses reveal that the local and global news channels were the main information sources. It is important to note that there was a significant concern among participants about the accuracy and reliability of the news received.

### Topic I2: People & Everyday life

The second topic centers on how the pandemic has affected people’s everyday life and personal health precautions. It encompasses discussions about COVID-19 symptoms such as fever, respiratory issues, risk exposure, and preventative measures like wearing masks and consulting healthcare providers (e.g., screening, physician consultations, cleanliness practices).

### Topic I3: Mandates & Mental health

This topic anchors on the impact of government mandates during the pandemic, including quarantine, work-from-home orders, travel restrictions, social distancing guidelines, and lockdowns. The implementation of these measures led to significant activity suspension, business closures, and mandatory home confinement. This situation resulted in feelings of isolation and exacerbated the mental health challenges among individuals.

In contrast to the relatively balanced distribution of topics in newspapers, we observed an uneven distribution of topics in interviews. This can be found from the probability of each topic, where Topic I1 (“News Broadcasting & Trust”) accounts 46% of the discussions, followed by Topic I2 (“People & Everyday life”) and Topic I3 (“Mandates & Mental Health”) with 28% and 25% respectively. This disparity reflects a more focused concern of participants on the trustworthiness of news information. In terms of the topics covered in the two data sources (i.e., newspapers and interviews), the results indicate that topics captured in interviews show a more personal experience and perspective on the pandemic, whereas newspapers cover a broader narrative, ranging from public, to political and economic.

Delving further into these topics, we find both convergence and divergence between the two data sources. But the divergence of topics seems to be the most dominant between the big-thick data. Specifically, newspaper articles predominantly focus on public health and vaccine development (Topic N1), whereas interviews emphasize more on the reliability of news. Second, newspaper articles center on vaccination processes and community health management (Topic N2), whereas interviews stress on personal health experiences and preventive measures, demonstrating a divergence between organized strategy and personal health concerns. Regarding societal impacts (Topic N3), newspaper articles look at the connection between health policies, societal adaptation and public responses, while interviews have more discussion about individual mental health impacts. Nonetheless, we also observe certain convergence between the two. For example, the topics of healthcare and community responses to the spread of the virus (Topic N4) and safety measures (Topic N5) in newspapers are also reflected in interviews (Topic I2 & I3), where people shared personal experiences, feelings, and actions taken for preventing the spread of virus.

Presidential election and economic impacts (Topic N6 & N7) are two unique topics to newspaper articles, which again underscores the character of newspapers in providing a broader and comprehensive narrative of the pandemic. Overall, these findings highlight the differences and similarities between newspapers and interviews, with newspapers providing a macroscope view of the pandemic and interviews offering a microscope perspective on personal experiences, emotions and concerns. Understanding such convergence and divergence can help us learn from past pandemic experiences, therefore enabling a more proactive approach to new emerging public health crises related to the spread of infectious diseases.

## Conclusion

The present study aimed to systematically explore the multifaceted nature of the pandemic’s impacts. By innovatively integrating “big-thick” data, we sought to uncover key topics surrounding the pandemic and vaccination, both in newspapers and individual narratives derived from interviews. Our study involved summarizing key topics from these two distinct data sources, interpolating them at different scales to highlight geographical variations, and cross-comparing them to underscore areas of convergence and divergence. We found that newspapers offered a macro-level perspective, providing a broader and more comprehensive view covering public health, policies and economics. While public health emerged as the most prominent topic, the overall distribution of topics in newspapers was relatively balanced.

In contrast, interviews provided a divergent view at the micro-level, focusing more intimately on individuals’ experience, emotions and concerns during the pandemic. The distribution of topics in interviews is relatively uneven compared to that of newspapers. A particularly notable finding from our study is the pronounced concern regarding the reliability of news information, as expressed in the interview narratives. However, both sources converge to highlight the profound impacts of the pandemic on daily life trajectories. For example, Topic N3 (Societal impact) and Topic I2 & I3 (People & Everyday life and Mandates & Mental health), all emphasize the physical and mental challenges people faced during the pandemic. The observed convergences and divergences underscore the importance of incorporating both macroscopic and microscopic perspectives in public health study, particularly during times of crisis.

Turning back to the reliability of the news as highlighted from the interview data (i.e., Topic I1) calls for further investigation into how the dissemination of information in mass media, or similar channels, shapes the behaviors and perceptions of people in real-world context. This aspect aligns closely with the principle of the Risk Information Seeking and Processing (RISP) model in communication research [36–37]. The model considers a range of individual factors, including information insufficiency, subjective norms, motivation, and perceived risk affective responses, to determine whether a person is likely to seek or avoid information related to a pandemic or hazard. Applying this model to future analyses could provide valuable insights into how information about infectious diseases is best communicated to the public, ensuring effective dissemination strategies that resonate with diverse audiences.

While our study provides valuable insights, it also has its limitations which can certainly be expanded. First, the geographical scope, while extensive, does not encompass all regional nuances in the U.S. Future studies could broaden this scope to explore topic variations across different states in the U.S or comparing between different countries. Additionally, while the GeoNames database used for geocoding has provided reliable foundation, geocoding evaluation remains an open area of research, particularly in accurately capturing geographical nuances at a more granular level. Future research could improve geocoding accuracy by adopting advanced neural network architectures for more detailed geocoding [e.g., 38–39]. Second, the demographic breadth of the interview participants may not fully represent the entire population’s experiences, thus including participants with different cultural backgrounds and socio-economic status is essential in future research. However, it should be noted that the interview process is extremely time-consuming and scalability will remain a challenge. Moreover, our study did not examine the interplay between mass media and individual perceptions. Future work could delve deeper into understanding how news media influences public behaviors, such as investigating health news framing and sentiments, and how the public responds to varying frames and sentiments. In terms of analysis, while LDA is a widely used technique for exploring topics, it has limitations such as inability to capture semantic relationships between words and fails for analyzing short-form text [40]. Therefore, future research might consider employing other NLP models, like BERTopic [41], to explore the depth of topic analysis.

Nonetheless, our study represents a novel effort in employing a “big-thick” data approach within the context of the COVID-19 pandemic. This novel methodological synergy has proven its power in uncovering insights into the complex and multifaceted nature of the pandemic’s impacts. Through the analysis of newspapers, we gained direct access to the practical issues and broad topics prevalent during the pandemic. In contrast, the analysis of interviews offered a contextual depth from the human-centric perspective, revealing the nuanced experiences and perceptions of individuals. By exploring both the areas of convergence and divergence in these topics, our study enhances the understanding of key issues that both disseminated to and resonate with the public. Overall, this study not only contributes valuable insights to the study of public health during the pandemic but also extends its implication to public health research at large, paving the way for future health communication and policy-making.

## Supporting information

S1 Fig The distribution of demographic characteristics of people participating in interviews or focus groups. (DOCX)

S2 Fig The distribution of newspapers from different newspaper sources. (*Note*: *there are 134 distinct newspaper sources*, *only the top 20 sources are displayed here*).
(DOCX)

S3 Fig The distribution of newspapers from different publishers. The red dash line indicates the average number of newspapers across all publishers.
(DOCX)

S4 Fig Coherence score analysis for optimal topic number selection in LDA Models for newspaper data and interview data. Left: coherence scores for newspaper data, showing the optimal number of topics at K = 7; Right: coherence scores for interview data, showing the optimal number of topics at K = 3.
(DOCX)

S1 Table The detailed description of built-in entity types in the Spacy package. (DOCX)
