# Supplementary material for: From print to perspective: A mixed-method analysis of the convergence and divergence of COVID-19 topics in newspapers and interviews
Source: PLOS Digit Health. 2025 Feb 5;4(2):e0000736. doi: 10.1371/journal.pdig.0000736 (PMC11798470; doi:10.1371/journal.pdig.0000736)
Supplement: S1 Fig — (DOCX) [file pdig.0000736.s001.docx]

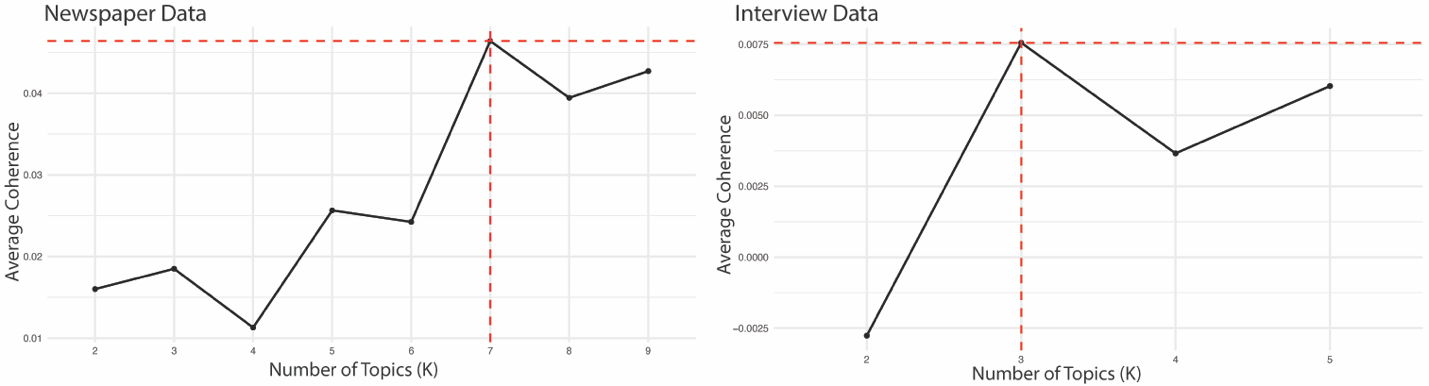


Figure S1. The distribution of demographic characteristics of people participating in interviews or focus groups.
