# Supplementary material for: From print to perspective: A mixed-method analysis of the convergence and divergence of COVID-19 topics in newspapers and interviews
Source: PLOS Digit Health. 2025 Feb 5;4(2):e0000736. doi: 10.1371/journal.pdig.0000736 (PMC11798470; doi:10.1371/journal.pdig.0000736)
Supplement: S2 Fig — (Note: there are 134 distinct newspaper sources, only the top 20 sources are displayed here). (DOCX) [file pdig.0000736.s002.docx]

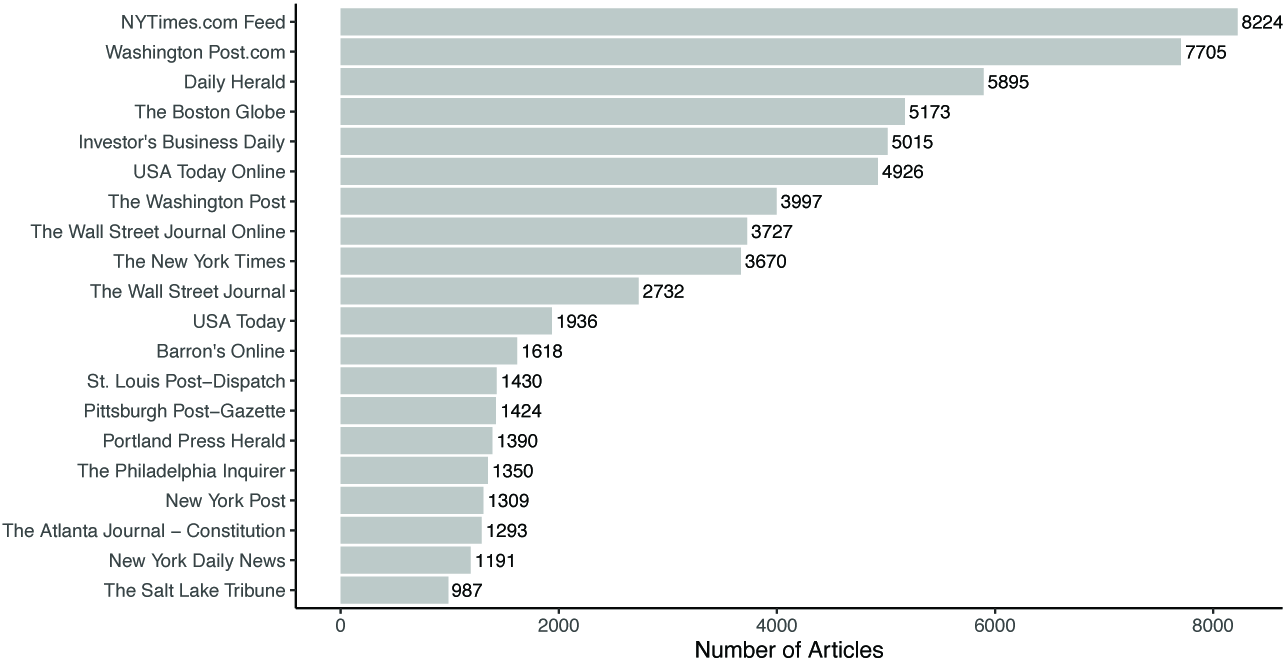


Figure S2. The distribution of newspapers from different newspaper sources. (*Note: there are 134 distinct* newspaper *sources, only the top 20 sources are displayed here*).
