# Supplementary material for: From print to perspective: A mixed-method analysis of the convergence and divergence of COVID-19 topics in newspapers and interviews
Source: PLOS Digit Health. 2025 Feb 5;4(2):e0000736. doi: 10.1371/journal.pdig.0000736 (PMC11798470; doi:10.1371/journal.pdig.0000736)
Supplement: S3 Fig — The red dash line indicates the average number of newspapers across all publishers. (DOCX) [file pdig.0000736.s003.docx]

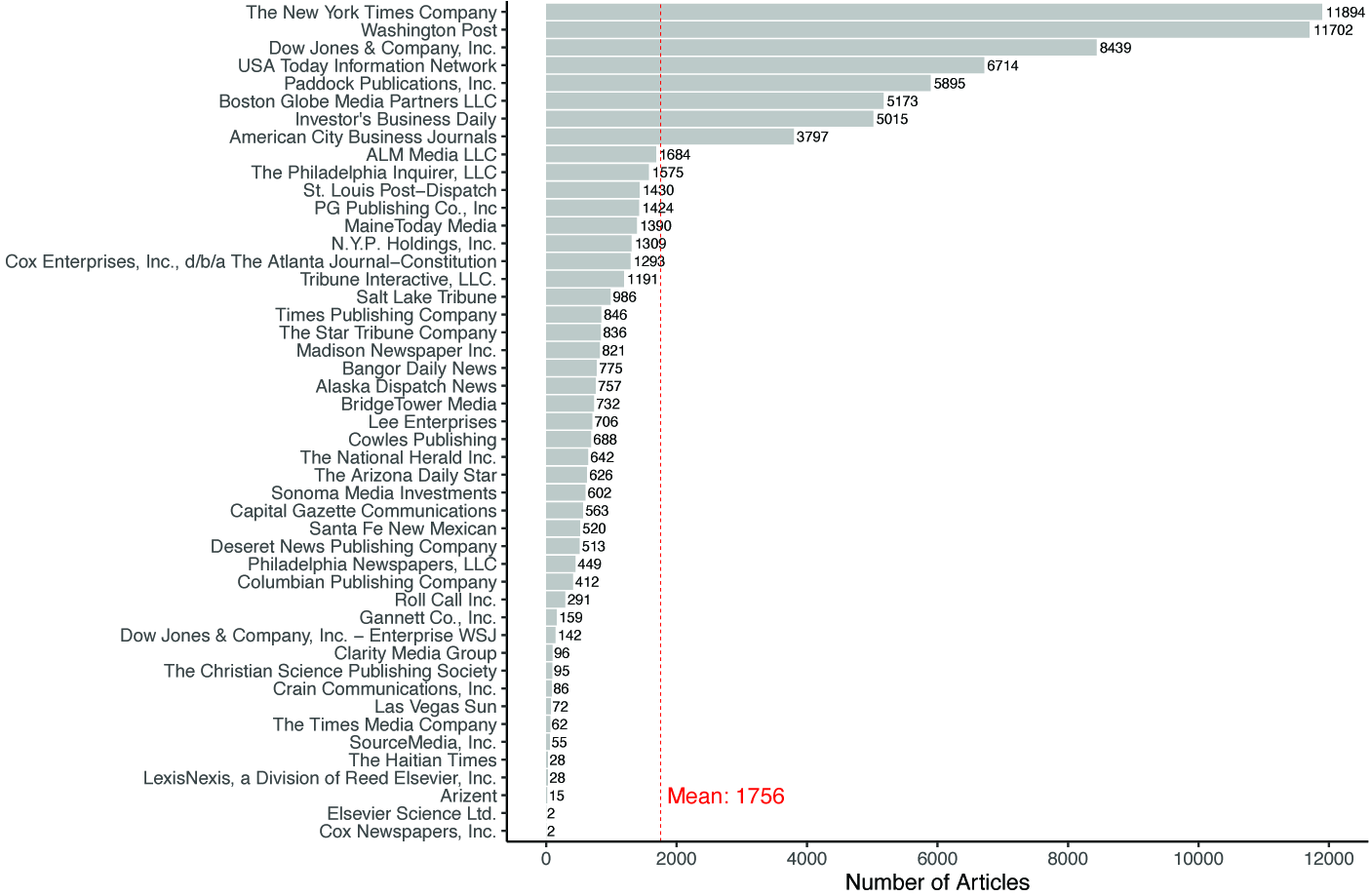


Figure S3. The distribution of newspapers from different publishers. The red dash line indicates the average number of newspapers across all publishers.
