# Supplementary material for: From print to perspective: A mixed-method analysis of the convergence and divergence of COVID-19 topics in newspapers and interviews
Source: PLOS Digit Health. 2025 Feb 5;4(2):e0000736. doi: 10.1371/journal.pdig.0000736 (PMC11798470; doi:10.1371/journal.pdig.0000736)
Supplement: S4 Fig — Left: coherence scores for newspaper data, showing the optimal number of topics at K = 7; Right: coherence scores for interview data, showing the optimal number of topics at K = 3. (DOCX) [file pdig.0000736.s004.docx]

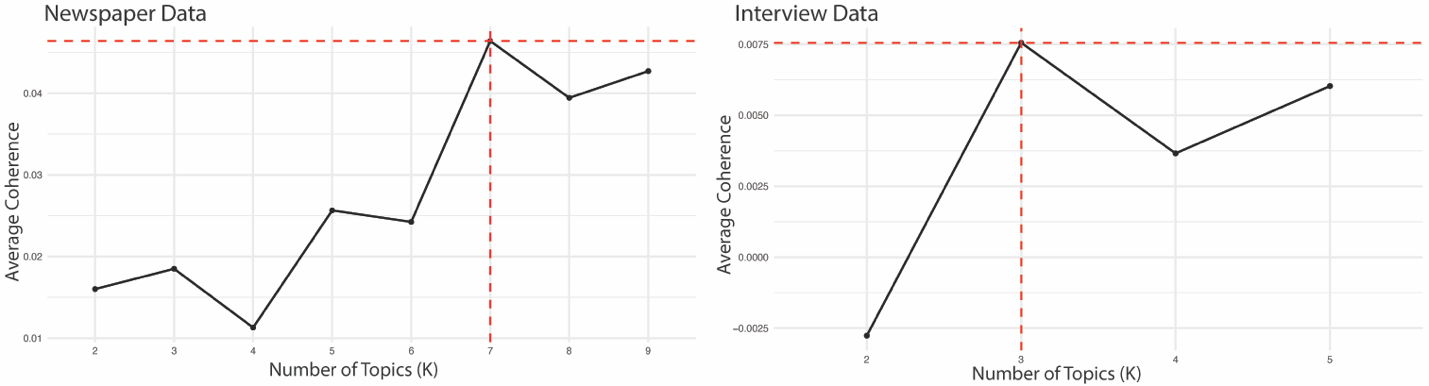


Figure S4. Coherence score analysis for optimal topic number selection in LDA Models for newspaper data and interview data. Left: coherence scores for newspaper data, showing the optimal number of topics at K = 7; Right: coherence scores for interview data, showing the optimal number of topics at K = 3.
