# Supplementary material for: From print to perspective: A mixed-method analysis of the convergence and divergence of COVID-19 topics in newspapers and interviews
Source: PLOS Digit Health. 2025 Feb 5;4(2):e0000736. doi: 10.1371/journal.pdig.0000736 (PMC11798470; doi:10.1371/journal.pdig.0000736)
Supplement: S1 Table — (DOCX) [file pdig.0000736.s005.docx]

Table S1. The detailed description of built-in entity types in the Spacy package.

| **Type** | **Description (examples)** |
| --- | --- |
| PERSON | People, including fictional |
| ORG | Companies, agencies, institutions, etc. |
| PRODUCT | Objects, vehicles, foods, etc (Not services) |
| LAW | Named documents made into laws |
| TIME | Times smaller than a day |
| QUANTITY | Measurements, as of weight or distance |
| NORP | Nationalities or religious or political groups |
| GPE | Countries, cities, states |
| EVENT | Named hurricanes, battles, wars, sports, events, etc. |
| LANGUAGE | Any named language |
| PERCENT | Percentage, including “%” |
| ORDINAL | “first”, “second”, etc. |
| FAC | Buildings, airports, highways, bridges, etc. |
| LOC | Name of politically or geographically defined location (cities, provinces, countries, international regions, bodies of water, mountains, etc.) |
| WORK_OF_ART | Titles of books, songs, etc |
| DATE | Absolute or relative dates or periods |
| MONEY | Monetary values, including unit |
| CARDINAL | Numerals that do not fall under another type |
